# A Systematic Review of Studies Describing the Effectiveness, Acceptability, and Potential Harms of Place-Based Interventions to Address Loneliness and Mental Health Problems

**DOI:** 10.3390/ijerph19084766

**Published:** 2022-04-14

**Authors:** Yung-Chia Hsueh, Rachel Batchelor, Margaux Liebmann, Ashley Dhanani, Laura Vaughan, Anne-Kathrin Fett, Farhana Mann, Alexandra Pitman

**Affiliations:** 1 Division of Psychiatry, University College London (UCL), London W1T 7NF, UK; yung.hsueh.20@alumni.ucl.ac.uk (Y.-C.H.); margaux.liebmann.20@alumni.ucl.ac.uk (M.L.); farhana.mann@ucl.ac.uk (F.M.); 2 Sussex Partnership NHS Foundation Trust, Worthing BN13 3EP, UK; rachel.batchelor.2015@live.rhul.ac.uk; 3 Bartlett School of Architecture, University College London (UCL), London WC1H 0AY, UK; ashley.dhanani@alumni.ucl.ac.uk (A.D.); l.vaughan@ucl.ac.uk (L.V.); 4 Department of Psychology, City, University of London, London EC1V 0HB, UK; anne-kathrin.fett@city.ac.uk; 5 Camden and Islington NHS Foundation Trust, London NW1 0PE, UK

**Keywords:** loneliness, mental health, built environment, nature, garden, community

## Abstract

Given the links between the built environment and loneliness, there is interest in using place-based approaches (addressing built environment characteristics and related socio-spatial factors) in local communities to tackle loneliness and mental health problems. However, few studies have described the effectiveness, acceptability, or potential harms of such interventions. This review aimed to synthesize the literature describing local community-based interventions that target place-based factors to address loneliness and mental health problems, informing the development of future public health approaches. We searched PsycINFO, Medline, and Embase using a structured search strategy to identify English-language studies evaluating the effectiveness, acceptability, and potential harms of place-based community interventions in addressing loneliness and mental health problems, both in general and clinical populations. Seven studies met the inclusion criteria, classified as evaluating provision of community facilities (such as clubhouses), active engagement in local green spaces, and housing regeneration. None were randomised trials. Quantitative and qualitative findings suggested promising effects and/or acceptability of six interventions, with minimal potential harms. There is a clear need for randomised trials or quasi-experimental studies of place-based interventions to describe their effectiveness in addressing loneliness and mental health problems, as well as complementary qualitative work investigating acceptability. This will inform future policy development.

## 1. Introduction

Loneliness is a distressing emotional experience representing a mismatch between actual and desired social connections [1] and is prevalent in the general population of many countries studied internationally [2]. Loneliness is now recognised as one of our most pressing public health issues [3] given its impact on physical and mental health and suicidal behaviour [2,4,5]. It is associated with premature mortality [6], including by suicide [7]. Loneliness also negatively affects the prognosis of mental health problems [8]. Although most research on loneliness has tended to focus on older adults, loneliness affects young people more than other age groups [9]. This is concerning given the rising prevalence of loneliness among young people [10] and the observation that half of mental health problems emerge before the age of 14 years [11]. This suggests that early intervention to address loneliness during adolescence could increase protective factors and improve adolescent wellbeing. This is even more pressing in the context of the COVID-19 pandemic, over which period loneliness, depression and anxiety have worsened [12,13], International data show that younger people and people with mental health problems reported the highest levels of loneliness [14], suggesting causal effects of loneliness on the mental health of young people [15].

It is becoming increasingly clear that the relationships between loneliness, social isolation (defined as an objective lack of social contacts), and mental health need to be considered in their wider built environmental contexts [16,17,18,19,20,21,22,23,24,25,26,27,28,29]. People living in remote areas experience poor transport links, reduced local activity choices, and poor digital connectivity. People living in urban areas may have richer social opportunities, but physical barriers (such as busy roads) and fears about safety can make social spaces inaccessible [30,31]. In each setting, such spatial factors have implications for loneliness, with evidence to support geographic variation in loneliness [32,33]. During the COVID-19 pandemic, the prevalence of the United Kingdom (UK) population reporting loneliness increased from 5% to 7.2%, with clear spatial patterning, such that people in rural areas reported feeling least lonely [34]. We know from published research that growing up in urban areas is a risk factor for mental health problems [35,36] and cognitive function [37,38], whereas growing up exposed to greenery is protective [39,40]. Generally, people who perceive their neighbourhoods as unsafe are more likely to report mental health problems [41]. Evidence also shows that people with a lower sense of belonging to their neighbourhoods and lower trust in its inhabitants feel lonelier [33,42] and that low neighbourhood social cohesion is associated with poorer mental health [35]. It is clear from this work that loneliness relates more to the physical character (density, design, or layout) of the local built environment and perceptions of it than the sheer number of people available to make contact with [43]. Intervening to modify features of the built environment could, in theory, increase opportunities for social connectedness, reducing loneliness and improving mental health. The neglect of this important physical dimension could explain why available intervention strategies to address loneliness yield only modest effects [44,45].

Such built environment characteristics, such as access to green spaces, buildings, parks, or street network connectivity, transport connectivity (with the wider area), and related socio-spatial factors (e.g., wealth of an area, social cohesion, ethnic homogeneity, and perceptions of neighbourhood safety) are hereafter referred to in this article under the umbrella term *place-based factors*. Interventions that address place-based factors might, for example, increase access to green space in immediate housing areas and locally (through creation of public parks or community gardens) or reduce the neighbourhood fear of crime (through improved street lighting). By promoting mixed land use and walkability, place-based interventions can increase opportunities for social interaction, inclusion, and cohesion, and in turn, it is possible that this might improve mental health [46]. However, few studies investigating the association between place-based factors and improvements in mental health have also measured impacts on loneliness [46]. Instead, we know from a systematic review of the evidence that some place-based interventions can improve mental health, and some can improve social isolation [47], but not whether they can achieve both or how. For example, the introduction of a neighbourhood renewal programme (involving external housing repairs, security, and road safety improvements) in the UK was associated with a significant decline in depression scores in both adults and children [41]. However, the mechanisms of this association are unclear without considering other factors such as improvements in social connectedness due to the creation of safer spaces. There is a clear gap in the literature regarding which place-based interventions have recognisable impacts on mental health and social connectedness, particularly those using randomised controlled trials (RCT) to assess effectiveness [48]. Whilst acknowledging the methodological difficulties of trialling such interventions given the complexity of the built environment [32], it is important for policymakers to make planning decisions based on evidence of effectiveness.

We aimed to synthesise the literature describing the effectiveness, acceptability, and potential harms of local community-based interventions that target place-based factors to address both loneliness and mental health problems, whether in the general population or in clinical populations. In doing this, we used a wide definition of loneliness to capture other dimensions of social connectedness, including social inclusion, social support, social capital, confiding relationships, and social connection [49]. This review was a response to policymakers’ call for evidence on this topic and is intended to inform the development of future public health approaches to reduce loneliness and mental health problems in general and clinical populations.

## 2. Materials and Methods

### 2.1. Search Strategy

We sought to include all potential qualitative and quantitative studies of interventions in communities that used place-based factors to address loneliness and mental health problems, irrespective of participants’ age and whether they had an underlying mental illness. We pre-registered the review protocol on the PROSPERO register of systematic reviews (CRD42021260165).

Three electronic databases (PsycINFO, Medline, Embase) were searched separately for any relevant studies from database inception until the end of May 2021. The search was conducted on 18 June 2021. As per our aim, search terms were linked to four concepts: exposure (“place-based factors”), intervention, outcome 1 (“loneliness”), and outcome 2 (“mental health problems or suicidality”). To determine search terms for each concept, we used team discussions representing a range of academic disciplines (built environment, social psychiatry, psychology) based on searches using Google Scholar. We used the Boolean operator “or” to combine all search terms within each concept. Our final search strategy (See Appendix A) combined all four concepts using the Boolean operator “and” in order to restrict the search to those studies investigating the impact of place-based factors on both loneliness and mental health problems. 

The first author then searched all three databases using the final set of search terms. While publication dates were not restricted, the searches were limited to English or Chinese language and human studies, with an additional limitation of full text studies. We also searched for any relevant studies through hand-searching through the reference lists of included studies, and by using the international email distribution lists of the Loneliness and Social Isolation in Mental Health research network and the Social Isolation & Loneliness Working Group. As Embase includes grey literature from conference proceedings and because our initial Google Scholar search process had identified no other unpublished studies, no further grey literature databases were used at this point.

### 2.2. Inclusion and Exclusion Criteria

Inclusion criteria were: (1) any empirical study design to assess effectiveness, acceptability, or potential harms of place-based interventions; (2) participants from any age group, with or without underlying mental health problems; (3) findings specific to interventions targeting place-based factors, such as built environment features or access to public facilities; (4) findings specific to interventions delivered in the participants’ local area or neighbourhood (e.g., walkable or a short bus journey away where transport required) and at the community level; (5) for interventional studies: quantitative outcome measures capturing validated or unvalidated measures of social connectedness (loneliness, social isolation, or social support) as well as validated or unvalidated measures of mental health; (6) for qualitative studies: findings describing the acceptability, perceived impact, or potential harms of such interventions. 

We therefore excluded studies evaluating interventions involving place-based interventions delivered outside the local area, and quantitative studies that captured solely measures of social connectedness or solely measures of mental health. 

### 2.3. Study Selection

All citations identified in the database search were imported into Covidence, a software package that facilitates systematic reviews, for further processing. After excluding duplicates, abstracts and titles were screened by the first author (Y.-C.H.) and a random sample of 10% of citations were independently screened by a second researcher (M.L.). Following abstract and title screening all eligible studies were subjected to a full text review by the first author for eligibility screening and a random sample of 10% of citations reviewed by a second researcher (M.L.). Disagreements at the title/abstract screening stage and at the full text review screening stage were discussed between the two researchers and with a third and fourth researcher (A.P. and R.B.) if no consensus was reached. Any relevant papers identified from reference lists whilst conducting the full-text screen were added to the included papers. 

### 2.4. Data Extraction

Relevant data were extracted by the first author into three proformas for quantitative studies (Table A1 in Appendix B), qualitative studies (Table A2) and mixed methods studies (Table A3), to capture domains agreed within the team as follows: author, publication year, setting, intervention, theory of change/likely mechanisms, sample size, participant characteristics (including mean age and proportion female), study design, outcome measures/analytic approach, key findings/themes, potential harms, and methodological limitations. For findings of quantitative and mixed methods studies, we reported all results instead of solely those relevant to our review questions to avoid missing context, but highlighted in the text those findings most relevant to our review question. 

### 2.5. Risk of Bias

To assess the quality of all eligible papers, we used the Mixed Methods Appraisal Tool (MMAT) [50] in view of its ease of use, good inter-rater reliability, and utility in systematic reviews of mixed study designs [51]. The MMAT contains five different categories: randomized controlled studies, non-randomized trials, quantitative descriptive studies, qualitative studies, and mixed methods studies. A range of criteria are listed for each study, scored as 1 if met, or 0 if unmet/unclear. The authors of the MMAT do not encourage reporting the quality of each study based on a calculation of an overall score from the ratings of each criterion, but instead suggest providing a more detailed presentation of the ratings of each criterion to better describe the quality of the included studies. The first author evaluated the quality of each included study, tabulating both detailed ratings and an overall proportion of criteria met. A second researcher (RB) conducted an independent evaluation of study quality, with disagreements discussed with a third researcher (AP). 

Our objective was not to exclude studies on the basis of quality assessment, but instead to present all findings in the context of their quality rating, to give more prominence to those using the most methodologically rigorous designs. 

### 2.6. Data Synthesis

We used the approach of narrative synthesis to summarise the findings, as we anticipated that included papers would use different study designs and that the number of quantitative studies would not be sufficient to conduct a meta-analysis. We sub-divided findings into categories based on intervention type; a subjective judgement of place-based factors as agreed upon by the research team. 

## 3. Results

### 3.1. Studies Identified

**Figure 1:**
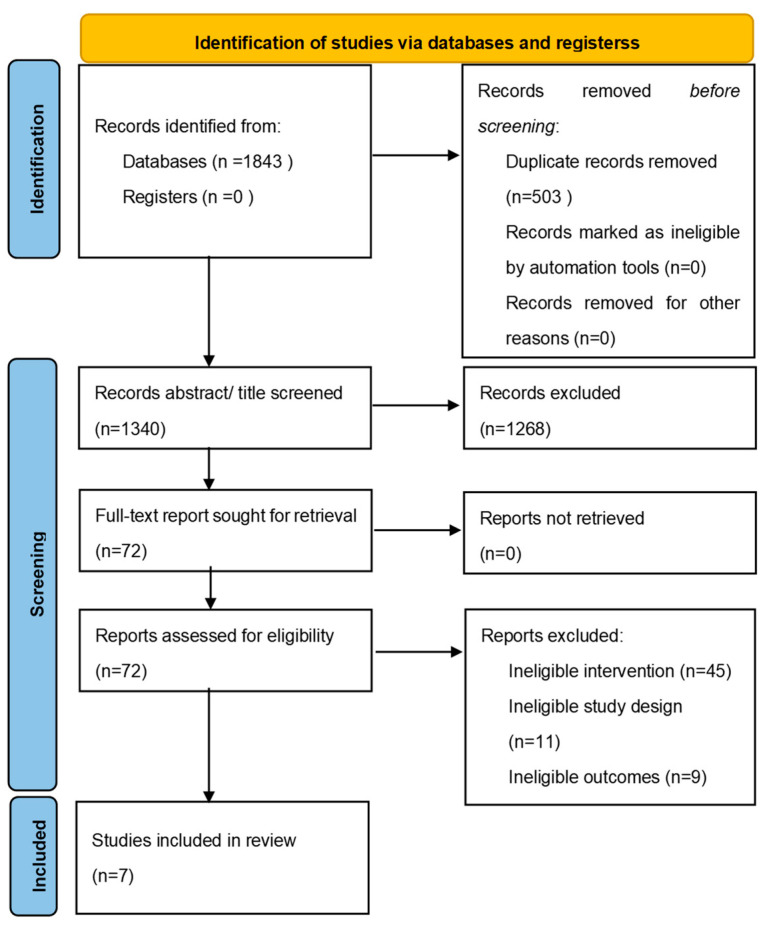
PRISMA flow diagram describing study selection.

Our database search identified 1843 citations, from which we removed 503 duplicates. We judged 1268 articles to be ineligible based on abstract/title screening. We then reviewed 72 full text articles and excluded 44 ineligible articles, identifying a total of seven studies eligible for inclusion (see Figure 1). All were studies published in peer-reviewed journals.

We observed substantial heterogeneity across study designs, settings, participants and interventions, and thus synthesised findings in a narrative review. Three studies were quantitative [49,52,53], two were qualitative [54,55], and two used mixed methods [56,57]. None of the studies were randomized controlled trials. Two of the quantitative studies used a pre-post study design [52,53] and one used a cross-sectional design [49]. One of the two qualitative studies collected data using face-to-face interviews [54], whilst the other used face-to-face interviews and fieldnotes on participants engaging in the intervention [55]. One of the two mixed methods studies collected data using a structured questionnaire followed by semi-structured interviews and group meetings to discuss the implications of findings [56]. The other mixed methods study collected wellbeing measures pre- and post-intervention and used a participatory process to record children’s views on their emotional wellbeing [57].

Settings ranged from Australia (n = 3), the US (n = 2), China (n = 1) and England (n = 1). Four studies sampled working-age adults (aged 18–60 years), two studies sampled older adults (aged 60–98 years), and one sampled schoolchildren (aged 9–15 years). Three studies sampled clinical populations with underlying mental health problems [54,55,57], while the remaining studies sampled people in the general population. Publication dates ranged from 2011 to 2020.

We noted a broad range of validated and unvalidated measures used to capture loneliness, social isolation and related concepts in the literature. These included loneliness, measured using the 3-item UCLA Loneliness Scale, and measures of social capital, social connectedness, social support and neighbourhood connectedness. A broad range of measures were also used to capture mental wellbeing, as measured through general mental health status, depression, anxiety, and psychological distress. However, no studies measured self-harm or suicide-related outcomes. 

### 3.2. Study Quality

The quality of studies was judged to be moderately high: three papers met 100% of quality criteria, three studies scored 80% and one met 60% of criteria (Table A4). No studies provided RCT evidence: the gold standard for evaluating effectiveness.

### 3.3. Results of Identified Studies

We summarised the findings of the eligible quantitative and qualitative studies by grouping them under three broad intervention categories: (i) provision of community facilities (n = 3), (ii) active engagement in local green spaces (n = 3) and (iii) housing regeneration (n = 1). In each case we inferred from the paper its theory of change (or likely mechanisms) to explain how the intervention was intended to bring about improvements in social connectedness and mental health. 

#### 3.3.1. Provision of Community Facilities

Three studies evaluated interventions delivered as local public facilities, namely park equipment, community canteens, and community clubhouses, as a focus for community activities such as exercise programmes and daily work tasks.

A quantitative before-and-after study by Levinger et al. (2020) set in Australia evaluated the Seniors Exercise Park program: an exercise and social support program designed to promote community wellbeing [52]. The intervention involved a 12-week physical activity programme for 95 older adults with a mean age of 73 years using the equipment provided in an outdoor Seniors Exercise Park. This was specifically designed for older people, to improve balance, joint movements, strength and overall mobility and function, with each session followed by time for socialising. The second part of the program involved 6 months of unstructured independent use of the Seniors Exercise Park equipment. The study compared pre- and post-intervention measures of loneliness, depression and wellbeing, with the primary outcome as level of physical activity at 9-month follow-up. Secondary outcomes included loneliness, social isolation, depression, and other psychosocial, mental health, and physical functioning measures at 9-month follow-up. Due to COVID-19 disrupting data collection, a decision was made to add analyses comparing baseline measures to those at 3-month follow-up. There were no significant differences in loneliness scores between baseline and 9-month follow-up, despite a significant reduction in loneliness scores at 3-month follow-up. Depression scores were significantly lower than baseline at 3 and 9-month follow-up. Social isolation scores did not differ at any time point. There were no significant differences in self-rated quality of life between baseline and 9-month follow-up, despite a significant improvement at 3-month follow-up. Other improvements at 9 months included physical activity level and function. Acceptability of the programme was captured through measuring adherence, which was 86% throughout the program. However, no figure was provided for adherence to the second part of the intervention; the 6-month maintenance phase involving access to the exercise park. For this study we judged that 80% of quality criteria in the MMAT scale were met, but had concerns about sampling bias with overrepresentation of females, limiting the generalisability of the findings to men. The overall findings suggest the potential for short-term effects on loneliness, quality of life, depression, and physical activity, but not social isolation, with benefits only sustained for depression and physical function. Longer-term follow-up on these measures would be valuable, and also allow investigation of the nature of social contacts (i.e., whether with other Seniors Exercise Park participants or other contacts local to the park). Qualitative work is required to understand the acceptability of the intervention and how older adults using the Seniors Exercise Park interact with other users both during the program and after it. For example, it would be important to understand how participants engaged in the 6-month maintenance phase of the intervention, and whether they continued to exercise with people they had met through the programme. A formal trial is also needed to ascertain effectiveness and investigate mechanisms, and to ascertain the contribution of being set in a green space.

A quantitative cross-sectional study by Wang et al. (2020) evaluated the community canteen scheme for 284 older people in a rural area of China [58]. This scheme is intended to address the recognised problems of food insecurity, nutritional status, and loneliness in older adults in China, and offers the opportunity for older adults to eat lunch and dinner together each day, for no or subsidised cost. In a cross-sectional study the authors described the association between use of council-funded community canteen services and a range of health and social outcomes, including social capital. It found that older people (mean age = 83) with access to a community canteen service reported significantly higher scores on social capital (*p* = 0.026) and satisfaction with life (*p* < 0.001) and better mental health (*p* = 0.038) than those with no access. We judged this study to meet 80% of MMAT quality criteria, as there was no adjustment for clustering or likely socio-economic confounders. Use of a cross-sectional design meant the authors were unable to infer causation. Additionally, villages sampled were in a relatively prosperous province, and the results may not be generalisable to less affluent regions of China. This study suggested positive impacts of community canteen services on the social capital, mental health and life satisfaction of older adults in rural China, but the intervention requires formal trialling and qualitative exploration of acceptability. Ideally this should involve investigation of the influence of walkability based on the location of community canteen services in relation to the villages’ geography, and associations with the home location of participants.

A qualitative interview study by Carolan et al. (2011) used semi-structured interviews to evaluate the acceptability of a clubhouse for people with mental health problems (including schizophrenia) provided in a rural community in a US mid-western state [54]. Clubhouses are intentional recovery communities where staff work alongside members, aiming to foster interpersonal relationships by facilitating ongoing mutual support. Interviewees were 20 adults aged 34 to 60 years who had attended the clubhouse at least three times a week within the last 6 months (of whom 12 had schizophrenia and 5 had affective disorders). The analysis of transcripts identified two key themes capturing the experiences of the clubhouse: clubhouse environment, and opportunities for growth. The first theme identified that the clubhouse provided a place for participants to be with others. It also provided structure to the day, with opportunities to volunteer for tasks involved in the running of the clubhouse, such as cooking, or clerical work, as an aid to transitioning into employment. Within this theme only one quote captured the interactions that club members had beyond the clubhouse, in the local area, but this was not explored further. The second theme identified the flexible environment and skills learned as a means by which participants were able to develop personal growth. This theme comprised three sub-themes capturing the clubhouse as a place to grow, to interact with others, and as a place where the staff were important in encouraging interactions, skill-building, promoting positive coping mechanisms and providing social support. By listening to and supporting clubhouse members, staff made them feel valued. Where clubhouse members took on new roles, such as giving talks about their experience and supporting others, they described having gained a sense of self-worth and empowerment. A key component of the clubhouse intervention was opportunities for socializing, in which to build relationships and create a sense of being included. No perceived disadvantages were reported by participants in this study, although the findings related to only one certified clubhouse. The study met all the quality domains and was therefore judged to be high quality. Overall, this study suggests a high degree of acceptability of providing a local clubhouse for people with mental health problems in promoting the building of social networks with others to facilitate recovery. It would be important to include in future evaluations an exploration of how the clubhouse builds social networks that are sustained beyond it, in the local area, and how other spatial characteristics of the clubhouse and its setting might facilitate this.

In summary, the findings of quantitative studies evaluating local community facilities for older adults in Australia and China found them to be associated with better social connectedness and mental health. However, effects on loneliness and quality of life may not be sustained beyond the delivery of the active intervention, and these findings may not be generalisable beyond high and middle-income countries. Formal trials with adequate follow-up are required to assess their effectiveness and potential harms in addressing loneliness and mental health problems. Findings of qualitative studies investigating the acceptability of clubhouses for working age adults with mental health problems suggest they are acceptable and perceived to be beneficial in improving social connectedness and mental health. Generally, a more formal description of the place-related characteristics of each setting would be beneficial in capturing these aspects of the intended outcomes of the intervention.

#### 3.3.2. Active Engagement in Local Green Spaces

Three of the eligible studies evaluated interventions involving active engagement in local gardening and conservation projects, including two community gardening projects and a school-based participatory gardening project.

A mixed methods study by Gerber et al. (2017) sampled 50 Bhutanese refugees living in the US to describe the cross-sectional association between engagement in community gardening and perceived social support and mental health, as well as qualitative interviews to measure acceptability [56]. It used quantitative survey methods to measure perceived social support, and symptoms of post-traumatic stress disorder (PTSD), anxiety, and depression and found that there were no differences in psychological symptoms between the 22 refugees engaged in the gardening project and the 28 refugees not involved. However, gardeners reported a higher level of social support (*p* = 0.017), particularly tangible support, such as help with meals and daily chores. Semi-structured interviews with eight gardeners and four non-gardeners probed issues such as the nature of social interactions whilst gardening (for gardeners), adjustment to life in the US, and perceived advantages and disadvantages of the community garden. Analysis of qualitative data found that those involved in the gardening project described receiving valuable practical social support, opportunities to build relationships and connection with others from their culture. They typically reported increased independence and self-efficacy, and identified the community garden as a place to share practical information and strengthen relationships. They also described a sense of empowerment through being able to save money and support their family. Some interviewees also reported improvements in mental health, physical health, and nutrition. A perceived disadvantage of the garden project was that it was run by refugee agencies, whereas if run by the participants themselves it might meet their needs better. Analysis of qualitative data from non-gardeners identified that lack of a consistent access to the garden was key barrier for them, but this was based on only four interviewees. The study met all quality criteria. However, it is important to note that in the meetings held to discuss findings, some interviewees expressed discomfort about voicing drawbacks to the intervention during individual interviews. This may have hampered disclosure of other perceived problems. There may have been some selection bias, as gardeners were recruited from established community gardens, with non-gardeners recruited through community meetings. Generally, the study highlighted the positive benefits of a refugee community gardening projects in fostering social support by strengthening relationships as well as sharing practical information, but no clear influence on mental health. Acceptability findings raised suggestions as to the need for a more co-produced approach to such projects. The intervention was undertaken in a local community garden, but no wider descriptors of the setting were provided. Future evaluations could consider the influence of local spatial factors on the acceptability and effectiveness of the intervention. 

A mixed methods study by Chiumento et al. (2018) evaluated the Haven Green Space gardening project, which involved 36 schoolchildren at three primary and secondary schools in England in monthly sessions (over the course of 6 months) working in teams to design a green space at their school supported by two horticulturists and a Child and Adolescent Mental Health Service (CAMHS) psychotherapist [57]. The target group were schoolchildren aged 9–15 years experiencing behavioural, emotional and social difficulties. The intention was to create a sense of connection with others, mastery of a new skill, including problem-solving experience, and a sense of giving back to the community. The study aimed to explore impact on mental wellbeing (using quantitative before-and-after approaches) and acceptability (using qualitative approaches). Comparison of pre-and post-intervention wellbeing scores showed no significant differences on mental wellbeing. Qualitative findings gathered through a workshop tool applied to children identified positive impacts of the intervention on emotional wellbeing, social networks and relationships, sense of belonging, having a valued role, knowledge and skills to make healthy choices, and having belief in personal capabilities and self-determination. The study met 60% of the quality criteria for quantitative studies, based on the unvalidated wellbeing measures used, the lack of adjustment for confounding factors (such as the transition from primary to secondary school for some participants), and the application of the workshop tool to children where it was designed for adults. This study suggested that group-based horticulture activities are perceived to benefit the emotional wellbeing and social connectedness of children, but findings may not be generalisable to those without behavioural, emotional and social difficulties. Additionally, the intervention’s ambition to improve connection to place was difficult to assess in the absence of a clear and consistent evaluation of the physical characteristics of the school green space, as well as the students’ responses to it.

A qualitative study by Whatley et al. (2015) described the acceptability of a community gardening and social participation intervention in Australia delivered to people with mental health problems in an inner-city area. The intervention involved 3 days per week of gardening activities, a weekly community kitchen, food enterprises, creative projects group, micro-enterprises, a weekly market, and a monthly community market. The study involved observing staff, participants and volunteers engaging in the Sprout community garden intervention between 2010 and 2011, and conducting semi-structured interviews with 13 participants and staff, to explore how it might influence social connectedness and wellbeing [55]. Analysis of field notes and interview transcripts identified three themes: creating community, a flexible environment that supports participation, and a learning environment. The first theme identified opportunities for building connections with each other and creating a sense of community through meaningful occupation, such as having meals or working together. The second theme described a flexible environment that encouraged participants to engage more by providing them with choices over what they participated in, which in turn helped them develop a sense of achievement through working with each other. The third theme described the creation of opportunities for personal growth, with participants gaining knowledge, taking on more responsibilities, and transitioning to coaching others. One perceived drawback identified by some participants was the anxiety created by the responsibility of running a stall in the market. However, this seemed to be mitigated by the social connections created through this opportunity. For quality assessment this study met all quality criteria. Generally, this study identified multiple advantages in social connection and wellbeing through participation in a socially inclusive gardening scheme for local people with mental health problems. However, the analysis did not explore whether the community gardening or the social components of the intervention were the more active ingredients, nor the role of local place-based factors in influencing social connections.

In summary, studies evaluating active engagement in local green spaces provide limited evidence to suggest that engagement in a Bhutanese refugee community gardening project is associated with better perceived social support but not better mental health. The evidence did not support engagement in community gardening for British schoolchildren as being associated with better mental wellbeing. However, qualitative findings supported the acceptability and perceived benefits of both these interventions, as well as of a community gardening intervention for people with mental health problems in urban Australia. We noted that study methodologies could not parse out the influence of nature-based aspects versus the interpersonal aspects of these collaborative social projects, and provided limited detail on local place-based characteristics. None of the studies in this category provided empirical evidence to support effectiveness in addressing loneliness and mental health problems, identifying a need for formal trials or quasi-experimental studies. Further work is also needed to investigate potential harms, such as excluding groups uninterested or unable to engage in gardening and/or social interventions set in a gardening context.

#### 3.3.3. Housing Regeneration

One eligible study evaluated a housing regeneration intervention using a before and after design. This quantitative study by Jalaludin et al. (2012) evaluated an urban renewal program in an area of 57 households in two streets in an area of southwest Sydney over the period 2009 to 2010. The program involved internal and external improvements to housing and social interventions such as community engagement activities, learning and employment initiatives, and provision of a community meeting place [53]. The study followed up adult householders living in the regenerated area to measure pre- and post-intervention scores on a range of validated neighbourhood perception measures (e.g., safety, aesthetics, walkability, connectedness and social capital), health risk behaviours, health status (including psychological distress), and health service utilisation. 

Comparing pre- and post-intervention measures the authors reported significant differences on specific items (relating to neighbourhood attractiveness, safety, and connectedness) but all *p*-values presented were uncorrected (despite the analysis pre-specifying a corrected *p*-value threshold) and were above the threshold for corrected *p*-values. We therefore inferred that there were no significant differences on any items pre- and post-intervention. The study met 80% of the quality criteria, as outcome measures were not validated, the sample size was small, and there was no control group outside the study area. Given reported *p*-values it was possible that the study was under-powered to detect any differences. Findings from one area of Sydney may also not be generalisable to other settings. 

This paper did not therefore provide evidence to support investment in an urban renewal program to improve social connectedness and mental health. Given the problems in trialling a housing renewal intervention (e.g., randomisation, taking sufficient account of contextual and temporal factors), more work is needed to enhance the measurement of place-based variables before and after investment in housing regeneration schemes. Alternatives to formal cluster RCTs, such as stepped wedge designs [59,60], may be more appropriate for evaluating the impact of large-scale investment in housing. This information will help policymakers ascertain the effectiveness and potential harms of spatial factors relating to housing regeneration interventions in addressing loneliness and mental health problems. 

## 4. Discussion

### 4.1. Main Findings

Our systematic review of studies summarised the evidence describing the effectiveness, acceptability and potential harms of local community-based interventions that target place-based factors to address both loneliness and mental health problems. Our strict inclusion criteria identified only seven studies, representing a balance of three quantitative, two qualitative, and two mixed methods studies, which overall we classified as: provision of community facilities, active engagement in local green spaces, and housing regeneration. None were randomised trials, so our review lacked trial evidence to support effectiveness, albeit acknowledging the challenges of designing trials of some of the interventions considered. We noted a limited range of interventions evaluated, with none addressing environmental factors that limit or promote access to social spaces, such as air pollution, lighting levels, transport connectivity, or communication networks. We also noted that many studies included in this review lacked nuanced descriptions of the local built environment context.

Of quantitative studies included, evaluations of the impact of interventions on loneliness and mental health identified evidence to support the use of local community facilities and of active engagement in green spaces in promoting the social connectedness and mental health of specific groups. However, cross-sectional designs or limited follow-up periods meant that there was no clear evidence that such benefits were sustained. These methodological problems, suggest a need for study designs that can investigate whether benefits are sustained and whether there is a need for booster sessions following interventions. There was no evidence to support an urban housing renewal program in improving social connectedness and mental health, but this study was likely to have been underpowered.

Of qualitative studies included, findings suggested that the interventions categorised under provision of community facilities and active engagement in green spaces were acceptable to participants. However, we lack evidence of the acceptability of housing regeneration interventions to residents, particularly given potential disruptions through building work. Only one potential harm was identified in this review as arising from a place-based intervention, namely the anticipatory anxiety of being given responsibility for running a market stall [54]. However, this was offset by the social connectedness benefits described. We therefore concluded that no significant harms were identified in interventions evaluated. 

None of the included studies described clearly the mechanisms by which place-based interventions might influence mental health through improvements in social connectedness, although qualitative findings provided clues as to such pathways. Instead, the theories of change we tabulated represent our own inferences. Medical Research Council (MRC) guidelines on the development of complex interventions advise that interventions specifying the underlying theory of change are more likely to be effective and sustainable because they consider local context and the stages of the causal pathway through which an intervention might achieve impact [61,62]. Further elaboration of the underlying theory of change for each intervention would help identify their active ingredients, and also assist in the future repurposing of effective interventions for other groups or settings. Many of the place-based interventions evaluated in this review involved a combination of place-based and social components, but with little consideration of which aspects exerted most influence on health and social outcomes. In order to gain a better understanding of the mechanisms of place-based interventions in improving health, two major advances are needed. Firstly, we need systematic collection of detailed place-based data before and after implementation of community-based interventions that target place-based factors, whether using objective or subjective measures of the built environment context [63]. This would allow us to parse out the contribution of compositional (individual characteristics of participants), contextual (specific features of the social and built environment) and collective (local values and traditions) variations in the studied area [64]. Secondly, we need adequately powered longitudinal studies that elucidate mechanisms by using causal inference-based approaches, as well as ascertaining how quickly place-based interventions impact social connectedness and mental health. Such work is critical for formal evaluation of the contribution of local place-based factors relative to the social or facilitation components, whether using trial designs or quasi-experimental designs based on observational data.

Together our findings suggest that there is a need for more quantitative and qualitative research investigating the effectiveness, acceptability and potential harms of a wider range of place-based interventions to address loneliness and mental health problems. Specific interventions categorised under use of local community facilities (Seniors Exercise Park program; council-funded community canteen services) and active engagement in green spaces (a refugee community gardening project) were suggested as most promising in benefiting mental health and/or social connectedness, and these might be prioritised for taking forward for formal trials. However, this evidence is drawn from a set of only seven eligible studies, and it seems important to collect more preliminary evidence of likely benefits from a broader range of place-based interventions. Beyond creative intervention development, the next stage for this field of research is to clarify the theoretical basis for place-based interventions by investigating mechanisms and identifying likely active ingredients, testing them for acceptability in a range of target groups. It will then be possible to consider the most feasible and methodologically sound approaches to evaluating them, acknowledging that RCTs may not always be appropriate for assessing their effectiveness and potential harms in addressing loneliness and mental health problems. 

### 4.2. Findings in the Context of Other Literature

Findings of other similar systematic reviews vary according to their specific inclusion and exclusion criteria, and this reflects wide variability in definitions of what constitutes a place-based intervention. Our own focus on local modifications of the built environment contrasted with the definitions used in other reviews. 

A 2018 systematic review of studies evaluated the effects of broadly defined place-based interventions (i.e., any physical change to the built environment) on mental health, isolation, and other outcomes, with studies measuring outcome at follow-up points between 1 year and 11 years [47]. However, unlike our review, it excluded studies in rural settings, interventions delivered in environments not accessible to everyone or inside buildings (e.g., private grounds, schools, hospitals), and studies where the main focus involved improvement or refurbishment to housing stock. Inclusion criteria did cover, however, broad interventions such as urban regeneration of a large urban area. Overall, it found no evidence to support an effect on mental health from urban regeneration or improving green infrastructure, but some evidence to support beneficial effects on social isolation two years after improving green infrastructure. Only one study in that review measured impact on both mental health and social isolation (using measures of perception of social ties with the community [65]), but had not been identified in our own review. This found no effect of a large-scale urban renewal project in deprived areas on any of these isolation measures, or on mental health. Unlike this 2018 systematic review, our own review did not exclude school-based interventions, resulting in our inclusion of one 2018 study evaluating a school-based gardening project on children’s emotional wellbeing, which found no impact on emotional wellbeing but a positive impact (measured qualitatively) on sense of belonging [57].

A 2021 systematic review of quantitative studies describing the acceptability and effectiveness of any interventions (delivered at individual, community or structural levels) to address loneliness and social isolation in young people found no studies evaluating place-based interventions [45]. It did not include the mixed-methods study in our review evaluating a school-based gardening project [57] because our review used a wider definition of loneliness to encompass other dimensions of social connectedness, and the school-based study had only captured loneliness in the qualitative component of the study, in terms of feeling involved and having a sense of belonging. 

### 4.3. Strengths and Limitations

Our systematic review is the first to synthesise the literature evaluating place-based interventions addressing loneliness and mental health problems at a community level in both general and clinical populations. At the review level, a key strength given the multidisciplinary nature of the study subject was our involvement of researchers from a broad range of disciplines (built environment, social psychiatry, psychology) and our team approach to developing a broad range of search terms to conduct a comprehensive review addressing a clear research question. We registered our protocol on PROSPERO and conducted our review following PRISMA guidelines. Our review combined both quantitative and qualitative findings, thereby providing a clear overview of the content and impact of interventions, and a deeper understanding of how they were experienced by participants. 

However, there were some limitations to the scope and execution of this review. As our search databases (i.e., Psych Info, Medline, Embase) were focussed on psychology and biomedical literature, potential studies published in the built environment literature may have been missed despite our efforts to use our research networks to seek out relevant studies. Additionally, in limiting our search to full-text and English or Chinese-language papers, we excluded the findings of abstracts and studies in other languages. Given the diversity of place-based factors, we acknowledge that our search terms were influenced by the subjectivity of researchers in our team, and this may have limited the range of studies identified. In focussing our scope deliberately on those interventions evaluated for their effectiveness in addressing both loneliness and mental health problems we are likely to have missed studies describing findings relating to the effectiveness of place-based interventions in improving only one or other of those categories of outcomes. These are potentially important in that they earmark interventions that could be evaluated for their impact on both categories of outcomes, and the mechanisms of any change. We acknowledge that findings from studies in very specific settings (and for specific populations) in Australia, the US, China and England may not be generalisable to other cultural settings, particularly low-income countries. Finally, there is an element of subjectivity in any systematic review, despite the efforts we made to conduct independent citation screening, data extraction and quality appraisal. Our narrative synthesis is likely to reflect our subjective appraisal of study findings, theories of change, and their implications, despite our interdisciplinary approach. 

### 4.4. Policy, Research and Clinical Implications

Given concerns about the health impacts of loneliness, a number of international governments have established ministers with responsibility for loneliness, taking a cross-departmental approach to review how changes to transport, urban planning, and community services might address loneliness by promoting social interactions and bringing together people who might otherwise not connect with each other [66]. In the UK, a government taskforce on loneliness has set out a number of recommendations based on a recognition of the socio-spatial barriers to connection in communities [67]. This suggested investing in community and social infrastructures to connect people, particularly in areas with higher levels of deprivation. Specific suggestions were made regarding loneliness-proofing all new transport and housing developments, and creating safe, welcoming and accessible green spaces, parks and gardens, and other facilities. However, this was with a clear awareness that the evidence to support this is lacking. A formal exercise to identify evidence gaps demonstrates a clear demand from policymakers for studies describing the influence of place on loneliness and mental health [68]. 

Our review provides evidence supporting the acceptability of a limited range of place-based interventions internationally, but also highlights the general absence of evidence describing the acceptability or effectiveness of a broader range of interventions, perhaps due to a lack of knowledge on mechanisms. It is possible that a RCT design is inappropriate for evaluating many place-based interventions, given the complexity of the built environment [32] and the difficulty of randomising contextual factors. However, alternative study designs are possible, such as analysing observational data from cohort studies to compare the effects of local exposure to place-based factors, using propensity scores to take into account group characteristics [69]. It will be important for such designs to explore the extent to which the inclusion of a facilitator (such as those supervising physical activity in the Seniors Exercise Park program [52]) is critical to the acceptability and effectiveness of the intervention. Where interventions targeting people with mental health problems are evaluated it will be important to avoid the tendency in the wider interventional literature on loneliness and mental health to focus on depression, but also to evaluate the acceptability and effectiveness of interventions for people with severe mental illness, personality disorders, and eating disorders [70].

Our review therefore identifies key directions for future research in this area, particularly in addressing the various methodological challenges identified by us in included studies, and highlighting the need for formal trials (or cohort studies) using validated measures of social connectedness and including suicidality among mental health outcomes. Methodological advances in the way that detailed spatial data are linked to longitudinal cohort data [39,63,71] would transform the way researchers might evaluate the health impacts of modifications to place-based factors. Findings from such observational studies would complement those from RCTs. Finally, given the high prevalence of loneliness in young people [10], evidence supporting the influence of neighbourhoods on child development [72], and the potential to intervene in childhood before mental health problems emerge [11], there may be grounds to prioritise the adolescent period when developing and evaluating place-based interventions to address loneliness. 

Our review suggests that there may be clinical advantages of certain place-based interventions for members of the general population and those with pre-existing mental health problems, particularly those involving use of community facilities and engagement in local green spaces. These findings are of interest for primary care clinicians practising social prescribing; a means of linking individuals who have psychological, social and/or practical needs with non-clinical services within their local community [73] as part of a wider care plan. They are also important in care planning within secondary mental healthcare. interventions described in our review presented opportunities for engagement in daily meaningful tasks and nature-based activities to promote skills-building, regulate emotions and cope with stress, provide social support, and create social connections. These were seen to have benefits on mental health for study participants in the general population, and were perceived to facilitate recovery among participants with mental health problems. It is therefore important that this review’s findings are disseminated to mental health professionals across the primary and secondary care system, promoting care planning that connects patients to their local communities.

## 5. Conclusions

Our review of only seven eligible studies from four countries provided some evidence to support the acceptability and perceived benefits of a limited range of six place-based interventions (categorised as community facilities and engagement in local green spaces) in improving loneliness as well as mental health problems, both in general and clinical populations. We found no evidence to support urban regeneration in addressing these outcomes. No studies included suicide-related outcomes, or tested mechanisms. All studies involved small samples, but no significant harms were identified. We identified no formal trials and therefore no formal evidence of effectiveness. This review therefore identifies a limited range of interventions likely to have beneficial effects on mental health and social connectedness, interventions that are likely to be acceptable to people in specific populations, and interventions needing further qualitative evidence of acceptability before proceeding to a feasibility trial. Investment is needed in intervention development and in methodologically rigorous evaluations of promising place-based approaches to promote social connectedness and mental health, as well as clarifying mechanisms of change.

## Data Availability

Not applicable.

## Appendix A. Search Strategy

### Appendix A.1. Concept: Place-Based Factors

Transit access OR transport OR transport connectivity OR public transport OR mobility OR commute OR road networks OR community severance OR barrier* OR broadband connectivity OR mobile networks OR digital connectivity OR noise pollution OR air pollution OR light pollution OR environmental pollution OR blue space OR green space OR greenblue space OR playgrounds OR skate parks OR recreational facilities OR resident associations OR subsidi?ed rent schemes OR youth cent* OR community cent* OR friendship bench OR urban design OR spatial factors OR street network connectivity OR social cohesion OR social deprivation OR neighbo?rhood perception OR neighbo?rhood deprivation OR ethnic homogeneity OR ethnic density OR population density OR neighbo?rhood crime OR high street OR library* OR park OR pub OR cafe OR community assets OR restorative niche OR nature OR urban public space OR land us* OR accessib* OR walkab* OR visibil OR urban OR suburban OR rural OR *crime OR community gardening.

### Appendix A.2. Concept: Intervention

Interven* OR treat* OR therap* OR program* OR trial OR prevent*.

### Appendix A.3. Concept: Loneliness

Loneliness* OR lonely* OR social isolation* OR confiding relationship* OR social exclusion* OR social network* OR social connect* OR social support adj5 (subjective or personal or perceived or quality) OR social relation* OR social capital* OR community engage* OR social engage* OR interact* OR isolate* OR perceived social isolation OR perceived social exclusion OR confiding relation* OR social defeat OR social withdrawal.

### Appendix A.4. Concept: Mental Health Problems OR Suicidality

Mental* health* OR mental disorder* OR mental* ill* OR mood disorder* OR psychiatr* OR depress* OR anxi* OR bipolar disorder OR bipolar OR post traumatic stress OR post?traumatic stress OR schizo* OR psychosis OR eating disorder OR anorexia OR bulimia OR personality disorder OR obsessive compulsive OR bab* blue* OR post-natal* OR psychological distress OR affective disorder OR adhd OR sadness OR Suicide OR overdose OR self-injurious behaviour OR suicidal behaviour OR automutilation OR drug overdose OR attempted suicide OR self destructive behaviour OR self-inflicted wounds OR self mutilation OR drug overdoses OR suicidal ideation OR self inflicted injuries OR non suicidal self injury OR self-harm* OR self?harm* OR self-injur* OR self?injur* OR self-mutilat* OR self?mutilat* OR suicide* OR self-destruct* OR self?destruct* OR self-poison* OR self?poison* OR overdos* OR self-immolat* OR self?immolat* OR self-inflict* OR self?inflict* OR auto-mutialt* OR auto?mutilat* OR NSSI.

Search dates (from and to): From database inception to 21 June 2021.

Restrictions: English, Chinese. No publication date restrictions.

## Appendix B

**Table A1:** Characteristics and findings of quantitative studies included in review (n = 3).

Study (Author & Year)	Country/Setting	Place-Based Intervention (and Category)	Theory of Change/Likely Mechanisms	Sample Size and Characteristics (Total Size, % Female, Mean Age)	Outcome Measure(s)	Study Design/Statistical Analysis	Key Findings	Potential Harms Identified	Methodological Limitations
**Provision of community facilities**									
Levinger et al., 2020 [52]	Australia; Elderly participants recruited from the general community in the suburbs close to the Seniors Exercise Parks in Melbourne, between October 2018 to November 2019	Seniors Exercise Park program: a 12-week structured supervised physical activity program using an outdoor exercise park, followed by a 6-month unstructured physical activity program (ongoing unsupervised access to the exercise park/twice a week exercise session with no formal structured group activity) Each structured exercise session was followed by morning/afternoon tea	Actively promote community wellbeing through the provision of a unique exercise and social support program in elderly people as well as the effects of sustained engagement in physical activity on physical, mental, social and health outcomes	- 95 elderly people (≥60 years) at baseline (mean age 73.0 ± 7.4; 82.1 % female) - follow-up data for 80 people to compare pre-post intervention scores (mean age 72.8 ± 7.5; 81.3 %) - 58 people took part in 9-month follow up	- Health-related quality of life using EQ-5D-5L/visual analogue scale (VAS); Mental wellbeing using WHO-5 Wellbeing questionnaire; Loneliness using 3-item UCLA Loneliness Scale; Depression using Geriatric Depression Scale (GDS-15); fear of falls using The Short Falls-Physical activity level using Community Healthy Activities Model Program for Seniors (CHAMPS) - Physical function using 2-minute walk test; step test; 4 m walk test - Falls Efficacy Scale International (Short FES-I) questionnaire; self-efficacy using The Self-Efficacy for Exercise (SEE); enjoyment using Physical Activity Enjoyment Scale (PACES); Social isolation and social support using Lubben Social Network Scale (LSNS6) - Fall risk assessment using The Fall Risk for Older People in the Community (FROP-Com)	Pre-post study design - Analysis of variance (ANOVA) repeated measures to compare scores on all measures at baseline and at 9-month follow-up - A separate ANOVA repeated measures examine the effect of the exercise program on all outcomes between baseline and 3 months/3 months and 9 months	- Significant increase in physical activity level after the intervention (*p* < 0.01, moderate to large effect sizes) - Significant improvements in all physical function measures (*p* < 0.01, small to large effect sizes), self-rated quality of life (*p* = 0.04, small effect size), wellbeing (*p* < 0.01, small effect size), fear of falls (*p* < 0.01, medium effect size), falls risk (*p* < 0.01, medium effect size), depressive symptoms (*p* = 0.01, small effect size), loneliness (*p* = 0.03, small effect size) - No significant changes in socialisation and self-efficacy for exercise outcomes (*p* > 0.05) - EQ-5D-5L: improvements in self-care (*p* < 0.01) and depression domains (*p* = 0.02) - 9 month follow up versus baseline: significant improvements in physical function (*p* < 0.05, moderate to large size effect); significant changes only in the health related quality of life mobility and self-care domains (*p* < 0.05, EQ-5D-5L), no changes in other domains; significant reductions in both fear of falls and falls risk (*p* < 0.01) - very few changes were observed between 3 and 9 months follow up - Improvement in depressive symptoms (Depression domain in Quality of Life Scale and GDS-15) - Slight improvement in loneliness but with no changes in social isolation/support (UCLA3/LSNS6 did not suggest experienced severe loneliness or lack of social engagement at baseline)	None	- COVID-19, associated restrictions and lockdown causing smaller sample in 9 months follow up (underestimation) - Level of physical activity was measured using self-reported questionnaire (CHAMPS), but has been widely used in research and suited for older Australian - Relatively high proportion of females, whereas males have been reported to have specific preferences and characteristic of exercise interventions
Wang et al., 2020 [58]	China; Elderly participants recruited from villages of Jinhua in Zhejiang, between July and October 2017	Community canteen services offered to older adults in 7 villages, compared with older adults from 7 closest villages without canteen services.	Recipients of the canteen service (canteen group; CG) would show significantly better health than the non-recipients (NCG)	Final sample size of 284 elderly people responded to the survey comprehensively - 140 participants with canteen services, 144 without - 148 females (52.1%) - Average age 83.07 ± 4.19, with a range of 75 to 98 years	- General mental health using Chinese version of General Health Questionnaire(GHQ-12) - Satisfaction with life using Chinese version of Satisfaction with Life Scale (SWLS) - Social capital using Social Capital Questionnaire (SCQ) developed by Yang with acceptable reliability and validity - Nutritional status using Chinese version of Revised Mini Nutritional Assessment Short-Form (MNA-SF)	- Cross-sectional design - Independent *t*-test to compare continuous variables - Chi-square test and Mann–Whitney test to compare categorical data - ANOVA and Fisher’s Least Significant Difference (LSD) test to compare nutritional differences among three groups (CG with government support and enterprise donation; CG with government support only; NCG)	- Adults in the CG had better mental health (mean = 1.39, SD = 1.95) and richer social capital (mean = 17.89, SD = 1.38) than NCG (GHQ: mean = 1.93/SD = 2.36; SCQ: mean = 17.48/SD = 1.64) - Nutritional status was not significantly different between CG and NCG, but was significantly different when considering funding sources and daily meal costs: ie those in the CG group with government support and enterprise donation (n = 40) had better nutritional status (mean = 13.28, SD = 1.32) - Those in the CG group had higher satisfaction with meals, self-evaluation of the meal nutrition, and regularity of meals compared to NCG - No significant differences in age, gender, marital status, educational level, or income between the CG and the NCG.	Not measured	- Cross-sectional study and only evaluated relationships at specific time points - Canteen service policies may be different for different provinces and result may not be applicable to poorer regions - Not measured confounding factors but only measured the benefits, not what people did not like - Self-report bias
**Housing regeneration**									
Jalaludin et al., 2012 [53]	Australia; Participants recruited from all 57 households in a suburb 45 km to the southwest of the Sydney central business district from December 2008 to April 2009	Urban renewal program conducted between April 2009 and August 2010, and in the two streets of established social housing - Internal upgrades: internal painting, replacement of kitchens, bathrooms and carpets where required, and general maintenance such as repairing water leakages, faulty windows and doors - External upgrades: property painting, new front and back fencing, new carports, letterboxes, concrete driveways, drainage, landscaping and general external maintenance - Social interventions: community engagement activities, learning and employment initiatives, and provision of a community meeting place.	The renewal program and its social components were intended to bring about improvement in social capital, social connectedness, a sense of community and in the economic conditions of residents.	- Total 42 participants followed up - Only 28 people completed both pre- and post-intervention surveys and were analysed (20 females; 86% aged 18–54 years)	- Psychological distress using Kessler Psychological Distress Scale (K10) - Questions about social connectedness, social capital, self-rated health, psychological distress and health risk factors as taken from the New South Wales Population Health Survey - Perceptions of neighbourhood safety, aesthetics and access to services within walking distance using the Neighbourhood Environment Walkability Scale (NEWS) - Adequate physical activity defined as a total of 150 min per week - Hazardous alcohol drinking defined as consumption of more than 2 standard drinks on any one day	Pre-post study design - Only included individuals who completed both surveys - Fisher-Freeman-Halton exact test to compare independent proportions - Paired chi-square tests (McNemar’s test) to compare paired proportions. Note that due to multiple testing, a *p* value threshold of 0.0013 was used, after applying the Bonferroni Correction but uncorrected exact *p*-values were presented throughout the manuscript.	Uncorrected *p* values presented suggested that there were no significant differences on any measures. The authors reported: no significant change in perceptions of neighbourhood aesthetics, safety or walkability, or in psychological distress and self-rated health. They reported a significant increase in the proportion of people reporting that there were attractive buildings and homes in the neighbourhood (18% versus 64%), and of feeling that they belonged to the neighbourhood (48% versus 70%), that their area had a reputation for being a safe place (8% versus 27%), they felt safe walking down their street after dark (52% versus 85%), and that people who came to live in the neighbourhood would be more likely to stay rather than move elsewhere (13% versus 54%). As the lowest uncorrected *p*-value presented was 0.0072, we infer that all corrected *p*-values showed no statistically significant findings.	None	- Small sample size - No comparison group (to take into account the influence of any changes occurring over that period due to factors other than the program) - Short follow-up - Results in the specific setting of this social housing neighbourhood may not be generalisable to other settings - Not possible to identify the active ingredients of the intervention, i.e., the urban renewal component versus the community engagement initiatives Use of uncorrected *p*-values throughout made it hard to interpret the findings, suggesting that no differences were significant

SD = standard deviation; CI = confidence interval.

**Table A2:** Characteristics and findings of qualitative studies included in review (n = 2).

Study (Author & Year)	Country/Setting	Place-Based Intervention (and Category)	Theory of Change/Likely Mechanisms	Sample Size and Characteristics (Total Size, % Female, Mean Age)	Study Design and Analytic Approach	Key Themes	Potential Harms Identified	Methodological Limitations
**Provision of community facilities**								
Carolan et al., 2011 [54]	United States; Participants recruited from a clubhouse in a rural community of a mid-western state	Clubhouse programme intended as a recovery community to foster interpersonal connections and support for individuals with mental illnesses	Based on a trans-ecological model that emphasizes the proximal relationships developed by individuals with persons and aspects of their environment	20 people (50% female) - Mean age 44 years (between 34 and 60 years of age) - 12 schizophrenia, 5 affective disorders - Inclusion criteria: members were involved with the clubhouse for at least six months and attended at least three times per week, within the last six months; ability to comprehend the consent process and coherently respond to interview questions to participate in a 60–90 min interview	- Semi-structured, open-ended interviews - Data coded using an adapted version of thematic analysis, based on a modified grounded theory approach (deductive and inductive methods)	Two overarching themes: Clubhouse environment: A place to be/clubhouse structure Opportunities for growth: A place to grow/a place to interact with others/Staff are important	None	- Involved only one clubhouse
**Active engagement in local green spaces**								
Whatley et al., 2015 [55]	Australia; Participants recruited from the staff, volunteers, participants, and support workers of a local community garden programme based in an inner city area of Melbourne.	Mind Sprout Supported Community Garden (Sprout): a local voluntary sector program offering 3 days per weeks to participants living in the local area who experienced mental ill-health, supported by staff, volunteers (some of whom had past mental health problems), and support workers from the participants’ mental health teams. The intervention comprised gardening activities, a weekly community kitchen, food enterprises, creative projects group, micro-enterprises, a weekly market, and a monthly community market.	The community garden model aimed to help enable occupational participation and social inclusion for people experiencing mental ill-health	- Observation of 13 study participants (4 staff, 5 project participants with mental health problems, 2 external support workers, 2 volunteers) observed over participant observation period - face-to-face interviews with 4 staff and 2 participants with mental ill-health to gain their views on how the intervention might effect change.	- Ethnography as research method, with data collected over the period November 2010 to January 2011 - Data included field notes taken during observation, a review of Sprout documentation, face-to-face interviews and photographs of the Sprout space/project. - Analysis occurred concurrently with data collection using open and focussed coding of field notes, collected documents and interview transcripts and of memos writing and mind mapping,	Three inter-related themes: Creating community: community connection as an outcome of participation Sprout: a flexible environment that supports participation Creating a learning environment	It was felt that having responsibility for working on the Sprout market stalls could create anticipatory anxiety for some participants, given the expectation of them running the stall smoothly. However, this seemed to be mitigated through the benefits gained in the social connections forged through the process of running the stall.	- Only a proportion of Sprout members were represented in this study, and the recruitment process was unclear - The first author’s role as an employee of Mind Australia may have influenced disclosure - the success of the programme in promoting future occupational participation and social inclusion was not explored as this was not part of the aims of the study.

SD = standard deviation; CI = confidence interval.

**Table A3:** Characteristics and findings of mixed methods studies included in review (n = 2).

Study (Author & Year)	Country/Setting	Place-Based Intervention (by Category)	Theory of Change/Likely Mechanisms	Sample Size and Characteristics (Total Size, % Female, Mean Age)	Means of Data Collection, Type of Data Collected	Analytic Approach	Key Findings (Effect Sizes, Key Themes, Efforts to Combine Findings from Quantitative and Qualitative Analysis)	Potential Harms Identified	Methodological Limitations
**Active engagement in local green spaces**									
Gerber et al., 2017 [56]	United States; Bhutanese refuges recruited from local community garden and Bhutanese community network	Local community gardening at two local community plots. Authors clarified that these were in an urban area, and that some participants had to use the bus to access the gardens, but all were local to residents.	- Gardening can boost connectivity and community strengths particularly for groups highly valuing communal functioning and cohesion - Expected outcome: Bhutanese gardeners would self-repot significantly fewer symptoms of distress and perceive higher levels of social support	- 50 people (62% female (n = 31)) - mean age = 44.5, SD = 15.01 - 56% non-gardeners (n = 28), 44% gardeners (n = 22)	Quantitative data: Structured questionnaires to collect cross-sectional data on: - symptoms of PTSD, anxiety, depression assessed by Refugee Health Screener-15 (RHS-15) in Nepali - Perceived social support using Medical Outcomes Study Social Support Survey (MOS SSS) - Somatization using the Patient Health Questionnaire-15 (PHQ-15), a 15-item self-report scale of somatization derived from the Patient Health Questionnaire, used previously with refugee populations, and translated into Nepali - Functional adaptation using Adapted Client Assessment Tool (ACAT) to describe current mastery or independence in 13 domains of functioning, orally administered Qualitative data: Semi-structured interviews to explore social support issues (such as the nature of social interactions whilst gardening), local acculturation (such as degree of adjustment to life in the US), and perceived advantages and disadvantages of the community garden. Group meetings were held with participants to study findings and explore implications.	Descriptive analysis of quantitative data. Adapted form of thematic analysis of interview transcripts for 8 gardeners and 4 non-gardeners, using the approach of Consensual Qualitative Research (CQR), which involved both deductive and inductive methods to code and interpret data. Focus groups were then used to discuss findings of analyses of the data above to gain feedback on results and consider implications in a culturally salient way.	Quantitative: - RHS indicated that distress was not significantly different between gardeners and nongardeners - MOS SSS: gardeners reported greater social support (a moderate to large effect size (d = 0.70; 95% CI 0.12, 1.27), subscales revealed that gardeners reported significantly more tangible social support (large effect size, d = 0.88, 95% CI 0.28, 1.45), medium effect size was observed for emotional/informational social support (d = 0.53, 95% CI −0.04, 1.09), small to medium effect for affectionate support (d = 0.34, 95% CI −0.23, 0.89), and positive social interaction (d = 0.31, 95% CI −0.26, 0.86) - PHQ-15 scores found to be not statistically significant but found a small to medium effect size (d = 0.36, 95% CI −0.21, 0.91) with gardeners having more somatic complaints - Exploratory correlational analyses: Age was additionally significantly related to perceived emotional/informational social support (r = −0.38, *p* = 0.007; 95% CI −0.60, −0.11), with perceived support decreasing with age; ACAT was significantly related to time in US (r = 0.30, *p* = 0.033, 95% CI 0.02, 0.53) Qualitative: - Key themes: General findings (results presented in all, or all but one), typical findings (more than half of the cases), variant findings (2 to 4 cases)	None	- Self-selection to group membership leading to gardeners significantly more likely to live in a house and lower medical bills - Randomization of assignments may adversely disruptive to the ethos of the established community - Elderly with physical limitations may have difficulty obtaining certain benchmarks - Small to moderate effect sizes may be missed, although sample size was sufficient for hypothesis testing - Under-reporting of adjustment difficulties as participants feeling discomfort voicing their problems in interviews
Chiumento et al., 2018 [57]	United Kingdom; Children recruited from 3 schools in the North West of England (two primary schools and one secondary school)	Haven Green Space school garden project, involving monthly sessions over the course of 6 months in which schoolchildren were supported at school by two horticulturists and a Child and Adolescent Mental Health Service (CAMHS) psychotherapist to work together in designing a green space	Promote positive mental, emotional and physical wellbeing of the children with the “Five Ways to Wellbeing” framework (Connecting with others; Being active; Taking notice of the local environment and of their feelings; learning horticultural skills and how to manage successes and failure; Giving back to the wider community	36 children (14 females) - children with behavioural, emotional and social difficulties - 2 primary schools (year 5 and 6, aged 9–12) and 1 secondary school (year 7 to 9, aged 12–15)	Quantitative data: Collection of pre- and post- intervention scores on the following measures for children - Mental wellbeing using Wellbeing check cards (based on the 7-item version of the Warwick Edinburgh Mental Wellbeing Sale) Qualitative data: collected over the course of 2 h workshops (pre- and post- intervention) by using the Mental Wellbeing Impact Assessment (MWIA) to plot data in a participatory way with children under the following three domains: (1) enhancing control (2) increasing resilience and community assets (3) participation and social inclusion. This qualitative tool aims to assess the potential impact of a specific policy, service, project or program on the mental wellbeing of a population, and was originally developed for adults.	Quantitative analysis: Statistical comparison of scores on pre- and post- intervention measures. Qualitative analysis: thematic analysis of data, with the coding process deductively driven by the MWIA themes. Separate analyses of the quantitative and qualitative data then converged in the discussion to triangulate findings.	Quantitative: -wellbeing scores not found to be statistically significantly different from pre- to post- intervention (although no test statistics were presented to support this statement) Qualitative: Analysis of MWIA plots produced during workshops identified pre- and post-intervention tendencies towards pro-social behaviour (“feeling involved”, “having a valued role”, “sense of belonging” and “social networks and relationships”) and emotional symptoms. The thematic analysis also found that factors relating to mental health and wellbeing were positively impacted, including “emotional wellbeing” and “self-help”.	None	- Lack of a valid control group and small sample size - Lack of validation of Wellbeing check cards in children or of the adaptation of the MWIA for children (in view of its - use of adult terminology) - Wellbeing check cards: limited in their intended ability to capture change over time

SD = standard deviation; CI = confidence interval.

**Table A4:** Quality assessment of included studies using MMAT.

Category of Study Designs	Methodological Quality Criterion	Levinger et al., 2020	Wang et al., 2020	Jalaludin et al., 2012	Carolan et al., 2011	Whatley et al., 2015	Gerber 2017	Chiumento et al., 2018
Screening questions	S1. Are there clear research questions?	√	√	√	√	√	√	√
	S2. Do the collected data allow to address the research questions?	√	√	√	√	√	√	√
Qualitative studies	1.1. Is the qualitative approach appropriate to answer the research question?	N/A	N/A	N/A	√	√	√	√
	1.2. Are the qualitative data collection methods adequate to address the research question?	N/A	N/A	N/A	√	√	√	√
	1.3. Are the findings adequately derived from the data?	N/A	N/A	N/A	√	√	√	√
	1.4. Is the interpretation of results sufficiently substantiated by data?	N/A	N/A	N/A	√	√	√	√
	1.5. Is there coherence between qualitative data sources, collection, analysis and interpretation?	N/A	N/A	N/A	√	√	√	√
**Quantitative non-randomized studies**	3.1. Are the participants representative of the target population?	×	√	√	N/A	N/A	√	√
	3.2. Are measurements appropriate regarding both the outcome and intervention (or exposure)?	√	√	×	N/A	N/A	√	×
	3.3. Are there complete outcome data?	√	√	√	N/A	N/A	√	√
	3.4. Are the confounders accounted for in the design and analysis?	√	×	√	N/A	N/A	√	×
	3.5. During the study period, is the intervention administered (or exposure occurred) as intended?	√	√	√	N/A	N/A	√	√
**Mixed methods studies**	5.1. Is there an adequate rationale for using a mixed method design to address the research question?	N/A	N/A	N/A	N/A	N/A	√	√
	5.2. Are the different components of the study effectively integrated to answer the research question?	N/A	N/A	N/A	N/A	N/A	√	√
	5.3. Are the outputs of the integration of qualitative and quantitative components adequately interpreted?	N/A	N/A	N/A	N/A	N/A	√	√
	5.4. Are divergences and inconsistencies between quantitative and qualitative results adequately addressed?	N/A	N/A	N/A	N/A	N/A	√	√
	5.5. Do the different components of the study adhere to the quality criteria of each tradition of the methods involved?	N/A	N/A	N/A	N/A	N/A	√	×
**% of criteria met**		80%	80%	80%	100%	100%	100%	60%

For mixed methods studies, the overall score was denoted as the lowest score awarded to any of the components of the mixed methods study (whether quantitative or qualitative) as per precedent, on the basis that the combined quality cannot be said to surpass the weakest quality component [51].
